# Efficacy and Safety of Surgical Techniques in the Management of Pulmonary Hydatid Disease: A Retrospective Cohort Study

**DOI:** 10.7759/cureus.82575

**Published:** 2025-04-19

**Authors:** Muhammad Imran, Muhammad Abid Khan, Ibrahim Janas, Mehran Ullah, Hidayat Ullah, Abdul Baseer, Fahad R Khan

**Affiliations:** 1 Thoracic Surgery, Lady Reading Hospital Medical Teaching Institute, Peshawar, PAK; 2 Cardiology, Lady Reading Hospital Medical Teaching Institute, Peshawar, PAK

**Keywords:** capitonnage, cystotomy, lung resection, postoperative morbidity, pulmonary hydatid disease, surgical outcomes

## Abstract

Objective

The primary objective of this study was to evaluate the effectiveness and safety profile of various surgical interventions used to manage pulmonary hydatid cysts, comparing patient outcomes such as postoperative morbidity, hospital stay duration, postoperative mortality, and complication rates across different surgical techniques.

Methods

This retrospective observational cohort study was conducted at the Department of Thoracic Surgery at Lady Reading Hospital, Peshawar, Pakistan, from January 1, 2023, to December 31, 2023. Patients with surgical treatment of pulmonary hydatid cysts were included. Surgical techniques ranged from lung-sparing procedures, such as cystotomy with capitonnage, enucleation, and pericystectomy, to more extensive resections, including wedge resection, segmentectomy, lobectomy, and pneumonectomy. Primary outcomes included postoperative morbidity, hospital stay duration, pain scores, and 30-day mortality. Statistical analysis was performed using analysis of variance (ANOVA) and Fisher’s exact test, with a significance threshold of p < 0.05.

Results

A total of 180 patients were included. The mean age of patients was 37.45 ± 11.28 years; 105 (58.33%) were men, and 75 (41.67%) were women. Cystotomy with capitonnage was performed in 97 patients (53.89%), showing the shortest hospital stay (9.4 ± 3.1 days) and the lowest morbidity (18 patients, 18.56%). Pneumonectomy was conducted in four patients (2.22%) and was associated with the longest hospital stay (17.8 ± 7.1 days) and the highest morbidity (three patients, 75.00%). Postoperative mortality occurred in three patients (1.67%), all of whom underwent extensive resections. The overall postoperative complication rate was 23.89% (43 patients), with chest pain in 99 patients (55.00%), cough in 81 (45.00%), and fever in 27 (15.00%). Statistically significant differences in hospital stay (ANOVA: p = 0.001) and morbidity (Fisher’s exact test: p = 0.01) were found, favoring lung-sparing techniques.

Conclusion

Lung-sparing procedures, particularly cystotomy with capitonnage, were associated with superior outcomes, including shorter hospital stays, lower morbidity, and no mortality, in this cohort. These findings support the prioritization of conservative surgical approaches in managing pulmonary hydatid disease (HD), especially in endemic and resource-limited settings.

## Introduction

Hydatid lung disease, primarily caused by the parasite *Echinococcus granulosus*, remains a significant health concern in many endemic regions worldwide, particularly in rural and agricultural communities. The condition commonly manifests as one or more pulmonary cysts that can lead to various respiratory complications, including cough, chest pain, dyspnea, and hemoptysis, which may become life-threatening if not managed in a timely and effective manner [1,2]. In endemic areas, hydatid cysts may occur in isolation or concurrently with hepatic hydatid disease (HD). Patients may remain asymptomatic until the cysts grow large enough to produce symptoms or complications. The current management of pulmonary hydatidosis primarily involves surgical intervention aimed at completely removing the cysts, alleviating symptoms, and preventing recurrence or complications such as rupture and secondary infection [3]. A range of surgical techniques are employed, from lung-sparing cyst removal procedures to more extensive lung resections. Common approaches include cystotomy with capitonnage (emptying the cyst and suturing the cavity); the enucleation of the cyst (careful removal without spillage), often followed by capitonnage; and pericystectomy (the removal of the cyst along with its fibrous pericyst). In more advanced or complicated cases, lung resection may be necessary. These include wedge resection (the removal of a small lung portion containing the cyst), segmentectomy or lobectomy (the removal of a larger anatomical section of the lung), and pneumonectomy (the removal of the entire lung) in the most severe cases [4].

Despite the availability of these options, there is ongoing debate regarding the optimal surgical approach that balances efficacy with minimal morbidity. Some advocate for lung-preserving techniques to maintain pulmonary function, while others argue that more aggressive resection may be warranted in cases of giant or multiple cysts to prevent recurrence. This uncertainty underscores the need to clarify the differences in patient outcomes associated with each surgical technique [5]. This study was designed to address gaps in understanding how different surgical interventions affect patient outcomes in pulmonary hydatid disease (HD). Specifically, we aimed to evaluate and compare postoperative results, such as complication rates, hospital stay duration, postoperative pain, and overall morbidity and mortality, across the spectrum of surgical techniques used for hydatid cysts. The objective of this study was to systematically compare the safety and efficacy of various surgical approaches in the management of parenchymal pulmonary hydatid cysts, using outcome measures such as hospital stay, morbidity, postoperative complications, and mortality. The goal was to generate clinically meaningful data that could help guide evidence-based surgical decision-making. Clarifying the outcome profiles of these surgical strategies is of significant clinical importance, as it can assist thoracic surgeons in selecting the most appropriate intervention for each patient. An evidence-based choice of surgical technique can improve prognosis, reduce healthcare costs associated with prolonged hospitalization or complications, and ultimately enhance patient quality of life [6]. By providing comprehensive insights into the effectiveness and safety of each approach, we aim to inform clinical decision-making and optimize the surgical management of pulmonary HD.

## Materials and methods

Study design and setting

We conducted a retrospective observational cohort study in the Department of Thoracic Surgery at Lady Reading Hospital, Peshawar, Pakistan. The study spanned from January 1, 2023, to December 31, 2023. This design was selected to efficiently utilize existing hospital records to evaluate surgical outcomes without the time and resource requirements of a prospective trial. The institutional setting is a high-volume tertiary care center located in an echinococcosis-endemic region, offering a robust patient population for evaluating surgical management strategies for pulmonary hydatid disease.

Ethical approval

This study was approved by the Ethical Review Board of Lady Reading Hospital Medical Teaching Institute, Peshawar, Pakistan (approval number: 1024/LRH/MTI/22). Given the retrospective nature of the study, the requirement for individual patient consent was waived. All data were anonymized to maintain patient confidentiality.

Population and sampling

All patients diagnosed with pulmonary hydatid disease (pulmonary echinococcosis) and managed surgically at our center between January 1, 2023, and December 31, 2023, were identified through the hospital’s electronic medical record system. A total of 180 patients met the inclusion criteria and were enrolled. Fifteen cases were excluded due to incomplete documentation or loss to follow-up. Cases involving the pleura, mediastinum, or extrapulmonary structures were excluded to maintain a uniform study population focusing solely on parenchymal pulmonary cysts. No additional exclusion criteria (e.g., age and comorbidities) were applied to preserve the real-world applicability of findings.

Intervention (surgical techniques)

All surgeries aimed for complete hydatid cyst removal with the maximal preservation of lung parenchyma. Surgical techniques were selected based on internationally accepted criteria, considering cyst size (>5 cm), number (single or multiple), anatomical location (central versus peripheral), and complications (e.g., rupture and infection) [7,8].

Cystotomy with capitonnage involved opening the cyst, evacuating its contents, and closing the residual cavity using purse-string sutures to obliterate dead space. This technique was typically used for uncomplicated and accessible cysts. Enucleation with capitonnage consisted of the gentle removal of the cyst from surrounding parenchyma, often aided by saline instillation to loosen adhesions, followed by the closure of the cavity with capitonnage. Pericystectomy entailed the removal of the fibrous capsule and cyst contents without excising lung tissue and was employed when the cyst was well-demarcated from adjacent structures.

In contrast, anatomical resections were performed for more complex presentations. Wedge resection with cystotomy involved excising peripheral lung tissue along with the cyst and was used in the presence of devitalized tissue or cyst wall thickening. Segmentectomy or lobectomy was undertaken when cysts were multiple, large, or centrally located, causing significant structural compromise. Pneumonectomy, the removal of an entire lung, was reserved for extreme cases of diffuse cystic involvement or failed lesser resections.

All surgeries were conducted under general anesthesia via standard thoracotomy. Intraoperative precautions included packing the operative field with hypertonic saline-soaked pads to prevent hydatid fluid spillage and minimize the risk of secondary echinococcosis or anaphylaxis. Any bronchial openings were sutured. Chest drains were placed in all patients. Capitonnage was not performed following anatomical resections.

Outcomes and data collection

The primary outcomes included postoperative hospital stay (in days), postoperative pain scores measured using the visual analogue scale (VAS) on postoperative day 1, and overall morbidity and mortality within 30 days of surgery. Postoperative morbidity was defined as any adverse event such as fever, wound infection, persistent air leak, empyema, or other complications during hospitalization or within 30 days post-surgery. Mortality was defined as death occurring during the index hospitalization or within 30 days postoperatively, regardless of cause.

Secondary outcomes included the incidence of chest pain, cough, fever, purulent sputum, allergic reactions (e.g., urticaria or bronchospasm), and hemoptysis.

Demographic, clinical, and operative data were recorded using a structured pro forma. Data collection was conducted by two independent reviewers who cross-verified the entries with source records for accuracy.

Statistical analysis

Statistical analysis was conducted using SPSS version 26.0 (IBM Corp., Armonk, NY). Continuous variables, such as patient age, cyst size, visual analogue scale (VAS) pain score, and the length of hospital stay, were summarized as mean ± standard deviation (SD). Categorical variables, such as gender, surgical technique, cyst location, and postoperative complications, were reported as frequency and percentage.

Comparative analyses were performed using the independent Student’s t-test or one-way analysis of variance (ANOVA) for continuous variables and Fisher’s exact test for categorical variables. A p-value of <0.05 was considered statistically significant. As this was a retrospective study with a fixed sample size, prior power calculation was not performed.

## Results

This retrospective cohort study included 180 patients who underwent surgical treatment for pulmonary hydatid disease at our center between January 2023 and December 2023. The mean age of the cohort was 37.45 ± 11.28 years. There were 105 (58.33%) male patients and 75 (41.67%) female patients. Regarding smoking status, 124 (68.89%) were non-smokers, and 56 (31.11%) were current or former smokers.

Hydatid cysts were more frequently localized in the right lung (102, 56.67%) than in the left (78, 43.33%). The mean cyst diameter was 7.4 ± 4.2 cm, with individual measurements ranging from approximately 1 cm to 20 cm. The most common comorbid conditions were hypertension (32, 17.78%), diabetes mellitus (26, 14.44%), and chronic obstructive pulmonary disease (COPD) (14, 7.78%) (Table 1).

**Table 1:** Baseline characteristics of participants Values are presented as mean ± standard deviation (SD) or number (n) with percentage (%), as appropriate COPD: chronic obstructive pulmonary disease

Characteristic	Value
Age (years), mean ± SD	37.45 ± 11.28
Gender: male, n (%)	105 (58.33%)
Gender: female, n (%)	75 (41.67%)
Smoking status: non-smoker, n (%)	124 (68.89%)
Smoking status: smoker, n (%)	56 (31.11%)
Hydatid cyst location: right lung, n (%)	102 (56.67%)
Hydatid cyst location: left lung, n (%)	78 (43.33%)
Cyst diameter (cm), mean ± SD	7.4 ± 4.2
Hypertension, n (%)	32 (17.78%)
Diabetes mellitus, n (%)	26 (14.44%)
COPD, n (%)	14 (7.78%)

Clinically, 103 (57.2%) patients presented with chest pain, 84 (46.7%) reported cough, and 31 (17.2%) had purulent sputum. Fever was documented in 24 (13.3%) patients. Allergic manifestations such as urticaria or bronchospasm were observed in 17 (9.4%), while hemoptysis was noted in four (2.2%). Interestingly, 29 (16.1%) patients were asymptomatic at presentation, with cysts discovered incidentally on imaging.

**Figure 1:**
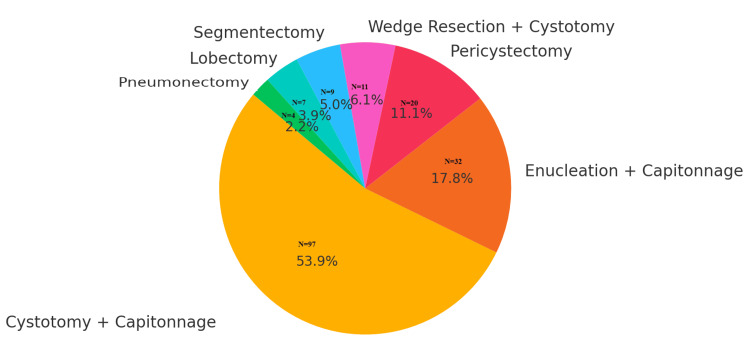
Distribution of surgical procedures performed among the 180 patients

The most frequently performed procedure was cystotomy with capitonnage (97, 53.89%), followed by enucleation with capitonnage (32, 17.78%) and pericystectomy (20, 11.11%). Lung resections included wedge resection with cystotomy (11, 6.11%), segmentectomy (nine, 5.00%), lobectomy (seven, 3.89%), and pneumonectomy (four, 2.22%). Figure 1 illustrates the distribution of surgical techniques employed.

Each segment of the pie chart (Figure 1) represents the proportion of patients who underwent a specific surgical procedure. This is also depicted in Table 2.

**Table 2:** Distribution of surgical techniques (N = 180) This table presents the frequency and percentage of different surgical procedures performed among the 180 patients treated for pulmonary hydatid disease. Values are reported as the number of cases (N) with corresponding percentages (%)

Surgical technique	n (%)
Cystotomy + capitonnage	97 (53.89%)
Enucleation + capitonnage	32 (17.78%)
Pericystectomy	20 (11.11%)
Wedge resection + cystotomy	11 (6.11%)
Segmentectomy	9 (5.00%)
Lobectomy	7 (3.89%)
Pneumonectomy	4 (2.22%)

The overall mean hospital stay was 10.8 ± 4.7 days. Postoperative morbidity was observed in 43 (23.89%) patients. Postoperative mortality occurred in three (1.67%) cases, all of which had undergone extensive lung resections. The mean pain score on day 1 post-surgery measured via visual analogue scale was 4.7 ± 2.3. A summary of the surgical outcomes is provided in Table 3.

**Table 3:** Primary surgical outcomes Postoperative hospital stay, pain scores assessed using the visual analogue scale (VAS), morbidity, and mortality in the overall cohort are summarized in this table. Results are reported as mean ± standard deviation (SD) or as absolute number (n) with percentage (%). No subgroup comparisons are presented in this table

Outcome	Value
Hospital stay (days), mean ± SD	10.8 ± 4.7
VAS pain score (0-10), mean ± SD	4.7 ± 2.3
Postoperative morbidity, n (%)	43 (23.89%)
Postoperative mortality, n (%)	3 (1.67%)

Focusing on secondary outcomes, we evaluated specific postoperative complications encountered in the cohort, as outlined in Table 3. The most common complication was postoperative chest pain (99, 55.00%), followed by cough (81, 45.00%), purulent sputum (29, 16.11%), and fever (27, 15.00%). Allergic reactions were reported in 16 (8.89%), while hemoptysis was seen in five (2.78%). All complications were managed conservatively. A detailed breakdown of postoperative complications is provided in Table 4.

**Table 4:** Postoperative complications Frequencies and percentages of individual postoperative complications. Data are descriptive. No statistical comparisons between subgroups were performed

Complication	n (%)
Chest pain	99 (55.00%)
Cough	81 (45.00%)
Fever	27 (15.00%)
Purulent sputum	29 (16.11%)
Allergic reaction	16 (8.89%)
Hemoptysis	5 (2.78%)

Comparative analysis revealed significant differences in hospital stay and morbidity across surgical groups (ANOVA, p = 0.001; Fisher’s exact test, p = 0.01). The shortest hospital stays and lowest morbidity were noted among those undergoing cystotomy with capitonnage. Pneumonectomy was associated with the longest hospitalization and highest complication rates.

We subsequently compared key clinical outcomes among the different surgical technique groups to evaluate the impact of surgical approach on postoperative recovery. Table 5 summarizes outcomes by surgical procedure.

**Table 5:** Comparison of postoperative outcomes by surgical technique Hospital stay differences were analyzed using one-way analysis of variance (ANOVA), and postoperative morbidity was assessed using Fisher’s exact test. An asterisk (*) denotes statistical significance (p ≤ 0.05). Cystotomy with capitonnage was associated with the shortest hospital stay and lowest complication rate, while pneumonectomy was linked to the longest hospital stay and highest morbidity SD: standard deviation

Surgical procedure	Hospital stay (days), mean ± SD	Postoperative morbidity, n (%)	Test statistic	P-value
Cystotomy + capitonnage (n = 97)	9.4 ± 3.1	18 (18.56%)		
Enucleation + capitonnage (n = 32)	10.7 ± 4.4	8 (25.00%)		
Pericystectomy (n = 20)	11.8 ± 5.2	6 (30.00%)		
Wedge resection + cystotomy (n = 11)	12.3 ± 5.0	4 (36.36%)		
Segmentectomy (n = 9)	13.2 ± 6.0	3 (33.33%)		
Lobectomy (n = 7)	14.5 ± 6.3	1 (14.29%)		
Pneumonectomy (n = 4)	17.8 ± 7.1	3 (75.00%)		
Overall comparison			F = 4.95; χ² = 13.28	0.001* (ANOVA); 0.01* (Fisher’s)

These findings underscore the superiority of lung-preserving surgical techniques, particularly cystotomy with capitonnage, in achieving favorable clinical outcomes with fewer complications and shorter hospital stays.

## Discussion

In this retrospective analysis of 180 patients undergoing surgical intervention for pulmonary hydatid disease, we investigated the efficacy and safety profiles of a spectrum of surgical techniques, ranging from lung-preserving procedures to more extensive resections. Our findings highlight that parenchyma-preserving procedures, particularly cystotomy with capitonnage, were associated with the most favorable postoperative outcomes, specifically shorter hospital stays, lower morbidity, and no reported mortality within that subgroup. In contrast, extensive resections, such as pneumonectomy, demonstrated higher morbidity and prolonged hospital stays, supporting the clinical rationale for minimizing the extent of surgical intervention when anatomically feasible.

These results are consistent with previous literature, which has repeatedly underscored the advantages of less invasive surgical options for managing pulmonary hydatidosis. Prior research has shown that cystotomy and capitonnage not only preserve pulmonary parenchyma but also minimize complications and long-term impairment of lung function [7]. Similarly, data from large case series by Burgos et al. [8] and Athanassiadi et al. [9] support the safety and efficacy of capitonnage as a reliable technique to obliterate residual cavities and prevent infections. Our study reinforces these conclusions and contributes real-world clinical data from an endemic, high-burden setting.

Despite the overwhelming preference for lung-preserving techniques, certain clinical situations necessitate extensive surgery. Pneumonectomy, although rare in our cohort (n = 4), was associated with the highest complication rate (75%), in line with the evidence indicating increased postoperative risks with major resections [10]. These risks are likely multifactorial, stemming from both the inherent trauma of removing an entire lung and the compromised baseline health of such patients. Intermediate procedures such as segmentectomy and lobectomy showed variable outcomes, and their morbidity profiles appeared to scale with the extent of resection. Factors such as cyst size, central versus peripheral location, and patient comorbidities likely influenced these results [11,12].

The stratified outcome data reinforce the need for individualized surgical planning, with decisions based on precise radiological evaluation, cyst characteristics, and functional pulmonary reserve. Notably, symptomatic morbidity, such as cough, chest pain, and purulent sputum, was frequently observed but managed conservatively. Fever and allergic reactions were transient and resolved without lasting sequelae, although they underline the importance of meticulous intraoperative handling to avoid cyst rupture and antigen exposure [3,13].

Allergic responses, including urticaria and bronchospasm, seen in approximately 9% of patients, further emphasize the necessity of stringent spillage prevention protocols, such as the application of hypertonic saline-soaked gauze and careful suctioning [13]. These practices are critical to prevent both immediate hypersensitivity and long-term recurrence due to intrathoracic seeding.

Postoperative symptoms such as persistent chest pain and cough, often reflective of pleural irritation, minor pneumothorax, or atelectasis, also underscore the need for optimal pain control and respiratory physiotherapy to enhance recovery [14]. From a healthcare system perspective, shorter hospital stays seen in patients managed with lung-preserving surgery have significant cost-reduction implications, especially in resource-limited, high-volume centers. Previous studies have indicated that such patients typically require less ICU care, return to daily function earlier, and pose fewer logistical burdens on surgical departments [15,16].

In terms of surgical hierarchy, cystotomy with capitonnage should be the first-line approach where applicable. For cases with extensive cyst burden, architectural lung distortion, or recurrent infection, segmental or lobar resections are justifiable. Our findings, and the existing literature, suggest that such decisions should be guided by cyst multiplicity, size, integrity, and any associated hepatic hydatidosis, even though we focused only on pulmonary involvement in this study [17].

Looking ahead, our results prompt important considerations for future research. Long-term follow-up data on recurrence, lung function, and quality of life would add critical value. Prospective studies incorporating postoperative spirometry, CT surveillance, and patient-reported outcomes would be essential for comprehensively evaluating the trade-offs between extensive and lung-sparing surgery. Further, while all patients in this study received albendazole, future investigations should compare its preoperative to postoperative impact, as prior research has hinted at its role in reducing recurrence and neutralizing intraoperative spillage [13].

Limitations

This study is not without its limitations. Its retrospective design inherently limits causal inference and is susceptible to selection bias, especially since surgical decisions were based on surgeon discretion and not standardized algorithms. Although we included consecutive cases to minimize sampling bias, unmeasured confounding may remain. Short-term follow-up further constrains our ability to assess recurrence and long-term complications. Additionally, although all patients had parenchymal pulmonary disease, we did not include extrapulmonary or mediastinal hydatid disease, limiting generalizability to broader thoracic hydatidosis.

Another methodological limitation stems from variability in documentation, a common issue in retrospective studies, which might have led to the underreporting of subtle complications or the misclassification of symptoms. Despite these constraints, our cohort size is substantial for this disease spectrum, and the multi-technique comparison adds depth to the understanding of real-world surgical outcomes.

In summary, our findings reaffirm that lung-sparing techniques, especially cystotomy with capitonnage, offer a safe, effective, and efficient treatment for pulmonary hydatid disease. When more extensive procedures are warranted, they must be planned with the full consideration of patient-specific factors and undertaken with rigorous perioperative support. Incorporating these principles into practice can significantly enhance clinical outcomes, resource utilization, and patient quality of life in endemic regions.

## Conclusions

Surgical management remains a cornerstone in the treatment of pulmonary hydatid disease, with our study demonstrating excellent outcomes and low mortality rates. Among the spectrum of surgical interventions, lung-sparing procedures, particularly cystotomy with capitonnage, were associated with the shortest hospital stays, lowest complication rates, and no mortality, reinforcing their role as the optimal approach when anatomically and clinically feasible. In contrast, patients undergoing more extensive resections, such as pneumonectomy, experienced significantly higher postoperative morbidity and prolonged hospitalization.

Our findings advocate for prioritizing lung-preserving surgeries to maximize safety, preserve pulmonary function, and enhance recovery. Thoracic surgeons should consider cystotomy with capitonnage as the first-line surgical strategy in suitable cases, reserving major resections for complex scenarios where disease eradication cannot otherwise be achieved. Adopting such a tailored approach can improve both patient outcomes and healthcare resource utilization, particularly in endemic and resource-constrained settings.
